# Childhood Emotional Abuse and Short-Term Stress Continuity Among University Students: The Role of General Music Preference

**DOI:** 10.3390/bs16091499

**Published:** 2026-08-26

**Authors:** Ke Qi, Lele Jiang, Shuhui Xu

**Affiliations:** 1 School of Music, Wenzhou University, Wenzhou 325035, China; 2 Department of Psychology, Wenzhou University, Wenzhou 325035, China

**Keywords:** childhood emotional abuse, general music preference, stress continuity, two-wave study, university students

## Abstract

We examined whether childhood emotional abuse was associated with short-term stress continuity among university students and whether general music preference was related to variation in the T1–T2 stress pathway. A two-wave prospective study with a three-month interval was conducted among 237 Chinese university students. Participants completed self-report measures of childhood emotional abuse, stress at both waves, and general music preference. Conditional process analysis was conducted while controlling for gender, academic year, and major type. Attrition and sensitivity analyses were also performed. Childhood emotional abuse was positively associated with both T1 and T2 stress, and T1 stress strongly predicted T2 stress, indicating short-term stress continuity. In the full sample, the interaction between T1 stress and general music preference was statistically significant, indicating that the T1–T2 stress association varied across levels of general music preference. However, the index of moderated mediation was not statistically significant because its bootstrap confidence interval included zero. Attrition analysis showed no significant difference in T1 stress between retained and lost participants. After music-major students were excluded, the associations among childhood emotional abuse, T1 stress, and T2 stress remained significant, whereas the interaction between T1 stress and general music preference was no longer significant. Childhood emotional abuse was associated with elevated and persistent stress across a three-month period among university students. The T1–T2 pathway should be interpreted as short-term stress continuity or carryover rather than as evidence of a distinct causal mediation process. Although general music preference was related to variation in stress continuity in the full sample, the nonsignificant index of moderated mediation and the sensitivity-analysis results indicate that this finding was not robust. The role of general music preference should therefore be considered preliminary and hypothesis-generating.

## 1. Introduction

Childhood emotional abuse is an important early risk factor for later psychological maladjustment. As a form of adversity occurring within relationships of trust and authority, it can undermine children’s sense of safety, worth, and emotional support, with lasting consequences for emotional development and self-evaluation (Bernstein et al., 1994; Brown et al., 2018; Schulz et al., 2014; Serafini et al., 2017). Prior research has linked emotional abuse and neglect to depression, anxiety, self-injurious behavior, and broader emotional difficulties across adolescence and adulthood (J. B. Gong & Chan, 2018; Shao et al., 2021; Xiao et al., 2023; Zhou & Zhen, 2022). One plausible pathway is that childhood emotional abuse may foster maladaptive emotion regulation, negative cognitive schemas, and heightened stress sensitivity, thereby increasing vulnerability to later stress (Alnassar et al., 2024; Heleniak et al., 2016; Musella et al., 2025; Yue et al., 2023).

The potential short-term persistence of stress associated with childhood emotional abuse is especially relevant in university populations. Developmental transitions, academic pressure, and changing social roles may amplify pre-existing vulnerability, making stress continuity particularly important during emerging adulthood. Empirical studies suggest that many university students report childhood trauma histories and that such histories are associated with elevated stress, depression, and anxiety symptoms (Bhattarai et al., 2023; Fu et al., 2018; Li et al., 2025). Although the association between childhood emotional abuse and later distress is well established, less is known about whether stress associated with childhood emotional abuse persists across short-term follow-up periods after baseline stress is taken into account. We therefore do not regard the association between childhood emotional abuse and later stress as the primary novelty of this research. Instead, we use this established association as the basis for examining short-term stress continuity among university students.

General music preference may represent one music-related individual difference associated with variation in stress continuity, although its role requires cautious interpretation. Music preference is relatively stable yet highly individualized, and previous research has linked it to emotional arousal, cognitive associations, social connectedness, and broader individual differences (Anderson et al., 2003; Buckley & Anderson, 2006; Greitemeyer, 2009; Juslin & Laukka, 2004; Koelsch et al., 2019; Rentfrow & Gosling, 2003; Ter Bogt et al., 2011). However, liking particular types of music is not equivalent to actually listening to music, engaging with music, or deliberately using music to regulate emotions. Music categories provide a general framework for describing individual preferences, but preference alone does not indicate how frequently or how long a person listens, the contexts in which listening occurs, or whether music is purposefully selected to cope with stress. General music preference is therefore best understood as a broad tendency to like music across different types rather than as a direct measure of music-based regulation.

Music preference, music exposure, and music-based coping are related but conceptually distinct aspects of musical experience. Listening frequency and duration reflect the extent of exposure to music, whereas listening motives and regulation strategies reflect the psychological functions that music serves. Research involving adolescents and young adults suggests that music may be used for distraction, solace, mood enhancement, emotional processing, tension reduction, and regulation of emotional intensity (Saarikallio & Erkkilä, 2007; Thoma et al., 2012). Music listening may also support arousal regulation, self-awareness, and social connectedness (Schäfer et al., 2013). Accordingly, individuals with similar music preferences may differ substantially in how often they listen to music and in whether they use music for emotional or stress-related coping.

The purpose for which music is used may be more directly related to stress outcomes than genre preference or listening frequency considered in isolation. In an ambulatory study, Linnemann et al. (2015) found that reductions in subjective stress and cortisol were particularly evident when participants listened to music for relaxation. Experimental evidence likewise suggests that stress-related outcomes may depend not only on the music itself but also on the regulation strategy adopted by the listener (Baltazar et al., 2019). At the same time, music-related emotion regulation is not necessarily adaptive in all circumstances. Some music-related regulation strategies have been associated with higher levels of depression, anxiety, and stress among young people, although the direction of these associations cannot be determined from cross-sectional evidence (Thomson et al., 2014). Taken together, these findings suggest that why, when, and how individuals listen to music may be as important as the types of music they prefer.

Distinguishing general music preference from actual music listening, music engagement, and deliberate music-based regulation is therefore important for understanding the relationship between music and stress. Music-related regulation may depend on preference, listening motives, frequency of exposure, cultural familiarity, situational context, and degree of engagement. Behavioral and neuroscientific research further indicates that music listening involves reward, prediction, and dynamic engagement, including anticipatory processing and responses to expected or unexpected musical events (Gold et al., 2023; Koelsch et al., 2019; Salimpoor et al., 2011). Daily-life research and meta-analytic evidence also support broader associations between music listening, emotion regulation, and stress reduction (Linnemann et al., 2015; Peters et al., 2024). Nevertheless, general music preference reflects an overall tendency to enjoy music and cannot substitute for direct assessments of listening behavior or music-based coping. Its association with stress continuity should therefore be regarded as a preliminary individual-difference relationship.

Music preference is also culturally embedded. Music categories, genre boundaries, and representative works vary across cultural contexts, and preferences are shaped by language, social experience, and cultural familiarity. The original music preference framework was developed primarily in Western contexts and may not fully capture the musical experiences of Chinese university students. Examining general music preference in this population therefore requires attention to culturally familiar music forms and to the meanings attached to different types of music. Cultural context is particularly relevant when interpreting whether a broad tendency to enjoy music is associated with psychological functioning across time.

Although substantial evidence links music to emotion and behavior, music-related individual differences have rarely been incorporated into two-wave models of stress following childhood adversity. Existing research has commonly used cross-sectional surveys to examine the psychological functions of music listening (Schäfer et al., 2013), laboratory experiments to assess immediate psychobiological responses to acute stress (Thoma et al., 2013), or ambulatory methods to investigate momentary or same-day associations between music listening and perceived stress (Linnemann et al., 2015). These approaches provide important evidence regarding the functions and short-term correlates of music listening, but they do not establish whether a broad individual difference such as general music preference is associated with the persistence or attenuation of stress across measurement waves. This question remains particularly relevant in the context of stress vulnerability associated with childhood emotional abuse.

Accordingly, we used a two-wave prospective design to address three questions. First, we examined whether childhood emotional abuse was associated with elevated stress across the two-wave assessment period. We treated this association as a preliminary empirical basis rather than as the primary contribution of the research. Second, we examined whether T1 stress statistically accounted for part of the association between childhood emotional abuse and T2 stress. Because stress was assessed repeatedly over a three-month interval, we interpreted this pathway as short-term stress continuity or carryover rather than as evidence of a distinct causal mediation process. Third, we explored whether general music preference was associated with variation in the strength of the T1–T2 stress pathway.

Based on previous research, we expected childhood emotional abuse to be positively associated with stress at both T1 and T2. We also expected T1 stress to predict T2 stress, reflecting short-term stress continuity. Finally, we explored whether higher general music preference was associated with weaker continuity between T1 and T2 stress. This exploratory question concerned broad music preference rather than listening frequency, listening duration, or purposeful music-based coping, and it did not assume that particular music genres have unique protective effects. Because the research was not preregistered and relied on observational two-wave data, the music-related analysis was considered theory-informed and hypothesis-generating rather than strictly confirmatory.

## 2. Method

### 2.1. Objectives

The primary objective of the present study was to examine whether childhood emotional abuse was associated with elevated stress at both T1 and T2 among university students and whether T1 stress accounted for part of the prospective association between childhood emotional abuse and T2 stress across a three-month interval. Because T1 and T2 stress represented repeated assessments of the same construct, this pathway was conceptualized as short-term stress continuity or carryover rather than as a distinct causal mediation process.

A secondary and exploratory objective was to examine whether the association between T1 stress and T2 stress varied as a function of general music preference and, consequently, whether the indirect association between childhood emotional abuse and T2 stress through T1 stress differed across levels of general music preference. Given the observational design and the preliminary nature of the music-related analysis, these findings were interpreted as hypothesis-generating rather than as evidence of a causal or protective effect.

### 2.2. Participants and Procedure

University students were recruited from 12 intact classes at one mainland Chinese university using class-based convenience sampling. The participating classes included eight classes from humanities, social sciences, psychology, and arts-related disciplines, including music, and four classes from science- and engineering-related disciplines. Classes were selected based on accessibility and the cooperation of course instructors, while seeking to include students from different disciplinary backgrounds. All students present in the selected classes were invited to participate. Participation was entirely voluntary, and only students who provided written informed consent completed the questionnaires. Data were collected online via Wenjuanxing (Questionnaire Star; Changsha Ranxing Information Technology Co., Ltd., Changsha, China) during regular class sessions and administered by psychology instructors at two time points. This procedure improved feasibility, minimized disruption to teaching, and helped ensure standardized administration. The study protocol was approved by the Institutional Review Board of Wenzhou University, and all participants provided written informed consent.

The first wave was conducted in October 2025 and yielded 488 questionnaires assessing childhood emotional abuse, current stress, and general music preference. Childhood emotional abuse was assessed at T1 because it was treated as a temporally prior risk factor, stress was assessed at both waves to examine short-term stress continuity, and general music preference was measured at T1 as a relatively stable individual-difference variable. The same 12 classes were approached again three months later, in January 2026, and stress was reassessed at T2. A total of 278 questionnaires were submitted at T2. The three-month interval was selected to allow short-term prospective variation in perceived stress while limiting excessive attrition.

Among the 278 respondents at T2, 254 had also participated at T1 and were therefore classified as retained for the attrition analysis, whereas 24 had not participated at T1 or had no identifiable T1 record. Among the original 488 T1 participants, 234 did not complete the T2 assessment and were classified as lost to follow-up. Of the 254 participants who submitted questionnaires at both waves, 17 were excluded from the final analytic sample because their records could not be reliably matched across waves owing to missing or incorrectly entered student identification numbers, because they had substantial missing data, or because their responses failed the data-quality checks. The data-quality criteria included invariant responding across questionnaire items (straight-lining) and incorrect responses to the attention-check item. The final matched analytic sample therefore comprised 237 participants. The T2 return rate among the original T1 cohort was 52.0% (254/488), and the final matched retention rate was 48.6% (237/488). Only complete and reliably matched cases were retained for the two-wave analyses.

The final sample included 79 males (33.3%) and 158 females (66.7%); gender was coded as 1 = male and 0 = female. By year of study, 23 participants were first-year students (9.7%), 156 were second-year students (65.8%), 55 were third-year students (23.2%), and 3 were fourth-year students (1.3%). For field of study, science and engineering, humanities and social sciences, and psychology were combined into a non-music-major group (*n* = 199, 84.0%; coded as 0), whereas music-major students were treated as a separate group (*n* = 38, 16.0%; coded as 1). Because participants were recruited from a single university and some academic years and fields of study were unevenly represented, the sample should be interpreted as institution-specific rather than representative of all Chinese university students.

### 2.3. Measures

#### 2.3.1. General Music Preference

General music preference was assessed using an adapted version of Rentfrow and Gosling’s (2003) 14-item music preference measure. The original instrument assesses preference for different music genres on a 7-point scale ranging from 1 (strongly dislike) to 7 (strongly like), with higher scores indicating stronger preference. To improve cultural comprehensibility among Chinese university students, the measure was modestly localized. Specifically, each music type was explained in Chinese according to its core stylistic features, and representative examples familiar to post-2000s Chinese university students were provided for each category. These examples were screened in a pilot test with 20 university students and were retained when more than half of the pilot participants judged them to be representative of the corresponding music type.

In the present study, the 14 items were averaged to create a general music preference score. This score reflects participants’ overall liking for different types of music. It was not interpreted as a measure of specific music-style dimensions, a unidimensional latent structure, or active music-based self-regulation. Cronbach’s alpha for the 14-item measure was 0.812, indicating good internal consistency. The adapted scale is provided in the Supplementary Materials.

#### 2.3.2. Childhood Trauma Questionnaire Short Form

Childhood emotional abuse was measured using the Chinese version of the Childhood Trauma Questionnaire Short Form (CTQ-SF) developed by Bernstein et al. (2003) and revised by Zhao et al. (2005). The CTQ-SF contains 28 items, including 25 clinical items and 3 validity items, covering emotional abuse, physical abuse, sexual abuse, emotional neglect, and physical neglect. Items are rated on a 5-point scale ranging from 1 (never) to 5 (always). The present study used the total score of the five emotional abuse items as the indicator of childhood emotional abuse. This choice was theory-driven. Because the focal research question concerned the long-term association between childhood emotional abuse and later stress, the emotional abuse subscale was analyzed separately rather than combining all trauma subtypes into a global adversity index. This strategy allowed closer alignment between the predictor and the proposed emotional-stress pathway. A sample item is “People in my family said hurtful or insulting things to me.” Cronbach’s alpha for the emotional abuse subscale was 0.785.

#### 2.3.3. Stress

Stress was assessed using the stress subscale of the Depression Anxiety Stress Scales-21 (DASS-21; Antony et al., 1998), Chinese version revised by X. Gong et al. (2010). The stress subscale includes seven items, such as “I found it hard to relax.” Participants rated how much each statement applied to them during the past week using a 4-point scale ranging from 0 (did not apply to me at all) to 3 (applied to me very much or most of the time). Higher scores indicate higher levels of perceived stress. This measure was selected because it captures core symptoms of tension, difficulty relaxing, and persistent stress reactivity, which are central to the present study. In addition, its brevity made it suitable for repeated administration in a longitudinal classroom-based design. In the present study, the reliability of the stress subscale was satisfactory, with Cronbach’s α values of 0.866 at Time 1 (T1) and 0.845 at Time 2 (T2).

### 2.4. Data Analysis

Normality was examined for T1 stress, T2 stress, general music preference, and childhood emotional abuse using one-sample Kolmogorov–Smirnov tests. All variables significantly deviated from normality, all *ps* < 0.001. Childhood emotional abuse showed the largest deviation, *D* = 0.246, whereas the deviations for the other variables were relatively smaller. Therefore, Spearman’s rank correlation coefficients were used to examine bivariate associations among the main study variables. This nonparametric approach provided a conservative preliminary assessment of associations before model testing.

We used Hayes’ PROCESS macro for SPSS (Model 14, version 3.0), implemented in IBM SPSS Statistics for Windows, version 20.0 (IBM Corp., Armonk, NY, USA), to examine the conditional process pattern. Specifically, we estimated whether childhood emotional abuse was associated with T2 stress through T1 stress and whether the association between T1 stress and T2 stress varied as a function of general music preference. Because T1 and T2 stress represented repeated assessments of the same construct across a three-month interval, we interpreted this pathway as short-term stress continuity or carryover rather than as evidence of a distinct causal mediation process.

All analyses controlled for gender, academic year, and major type. Gender was coded as 1 = male and 0 = female; major type was coded as 1 = music major and 0 = non-music major. Continuous predictors were mean-centered before analysis to facilitate interpretation of interaction terms. Indirect and conditional indirect effects were evaluated using bias-corrected bootstrap procedures with 5000 resamples. Effects were considered significant when the 95% bootstrap confidence interval did not include zero.

A sensitivity power analysis was conducted using G* Power for the focal interaction effect in the regression model predicting T2 stress. Using a linear multiple regression model testing the increase in R^2^, with *N* = 237, α = 0.05, statistical power = 0.80, one tested predictor, and seven total predictors, the minimum detectable incremental effect size was *f^2^* = 0.0334. Thus, the sample was capable of detecting an interaction effect of approximately this magnitude or larger with 80% power. This analysis was specific to the focal interaction term and did not constitute a power analysis of the overall moderated mediation effect.

To assess whether attrition introduced systematic bias, we compared participants retained in the follow-up sample (*n* = 254) with those lost to follow-up (*n* = 234) on T1 stress. An independent-samples *t* test showed no significant difference between the retained group, *M* = 1.75, *SD* = 0.50, and the attrition group, *M* = 1.72, *SD* = 0.50, *t*(482.07) = −0.60, *p* = 0.547. This result suggests that attrition was not systematically associated with baseline stress levels, although selective attrition on unmeasured variables cannot be fully ruled out.

We plotted the simple slopes using R version 4.6.0 (R Foundation for Statistical Computing, Vienna, Austria) and the ggplot2 package (version 4.0.2) to illustrate the interaction between T1 stress and general music preference in predicting T2 stress. Low and high levels of general music preference were defined as one standard deviation below and above the mean, respectively.

Given the two-wave observational design, the absence of intervening event measures, the reliance on self-report data, and the reduced matched panel after attrition, the analyses were interpreted as theory-informed and exploratory. Accordingly, the findings should be viewed as evidence of short-term prospective associations rather than as causal, clinical, or intervention effects.

## 3. Results

### 3.1. Descriptive Statistics and Spearman Correlations

We found that T1 stress was positively correlated with childhood emotional abuse, ρ = 0.358, *p* < 0.001, and T2 stress, ρ = 0.671, *p* < 0.001. Childhood emotional abuse was also positively correlated with T2 stress, ρ = 0.309, *p* < 0.001. General music preference was not significantly correlated with T1 stress, ρ = −0.035, *p* = 0.588; childhood emotional abuse, ρ = −0.030, *p* = 0.641; or T2 stress, ρ = −0.039, *p* = 0.553. These results indicated that students who reported higher levels of childhood emotional abuse also reported higher stress at both T1 and T2, and that higher T1 stress was associated with higher T2 stress. General music preference was not significantly associated with stress or childhood emotional abuse at the bivariate level. Descriptive statistics and bivariate correlations are presented in Table 1.

### 3.2. Conditional Process Analysis

Hayes’ PROCESS Model 14 was used to test whether the indirect association between childhood emotional abuse and T2 stress through T1 stress varied as a function of general music preference, while controlling for gender, academic year, and major type. All continuous variables were mean-centered before the analysis.

In the full sample (*N* = 237), childhood emotional abuse significantly predicted T1 stress, *b* = 0.3542, *SE* = 0.0608, *t* = 5.83, *p* < 0.001, 95% CI [0.2344, 0.4739]. In the model predicting T2 stress, T1 stress was a significant positive predictor, *b* = 0.5881, *SE* = 0.0543, *t* = 10.83, *p* < 0.001, 95% CI [0.4812, 0.6951]. The direct association between childhood emotional abuse and T2 stress also remained significant, *b* = 0.1221, *SE* = 0.0536, *t* = 2.28, *p* = 0.0237, 95% CI [0.0164, 0.2278].

The interaction between T1 stress and general music preference was statistically significant, *b* = −0.1110, *SE* = 0.0437, *t* = −2.54, *p* = 0.0117, 95% CI [−0.1972, −0.0249]. Thus, in the full sample, the association between T1 stress and T2 stress varied as a function of general music preference.

The conditional indirect association between childhood emotional abuse and T2 stress through T1 stress was statistically significant at each examined level of general music preference. The estimated indirect association was 0.2476 at a low level of general music preference (−1 SD), Boot*SE* = 0.0549, 95% BootCI [0.1538, 0.3698]; 0.2083 at the mean level, Boot*SE* = 0.0452, 95% BootCI [0.1319, 0.3076]; and 0.1690 at a high level of general music preference (+1 SD), Boot*SE* = 0.0417, 95% BootCI [0.0996, 0.2649].

**Figure 1:**
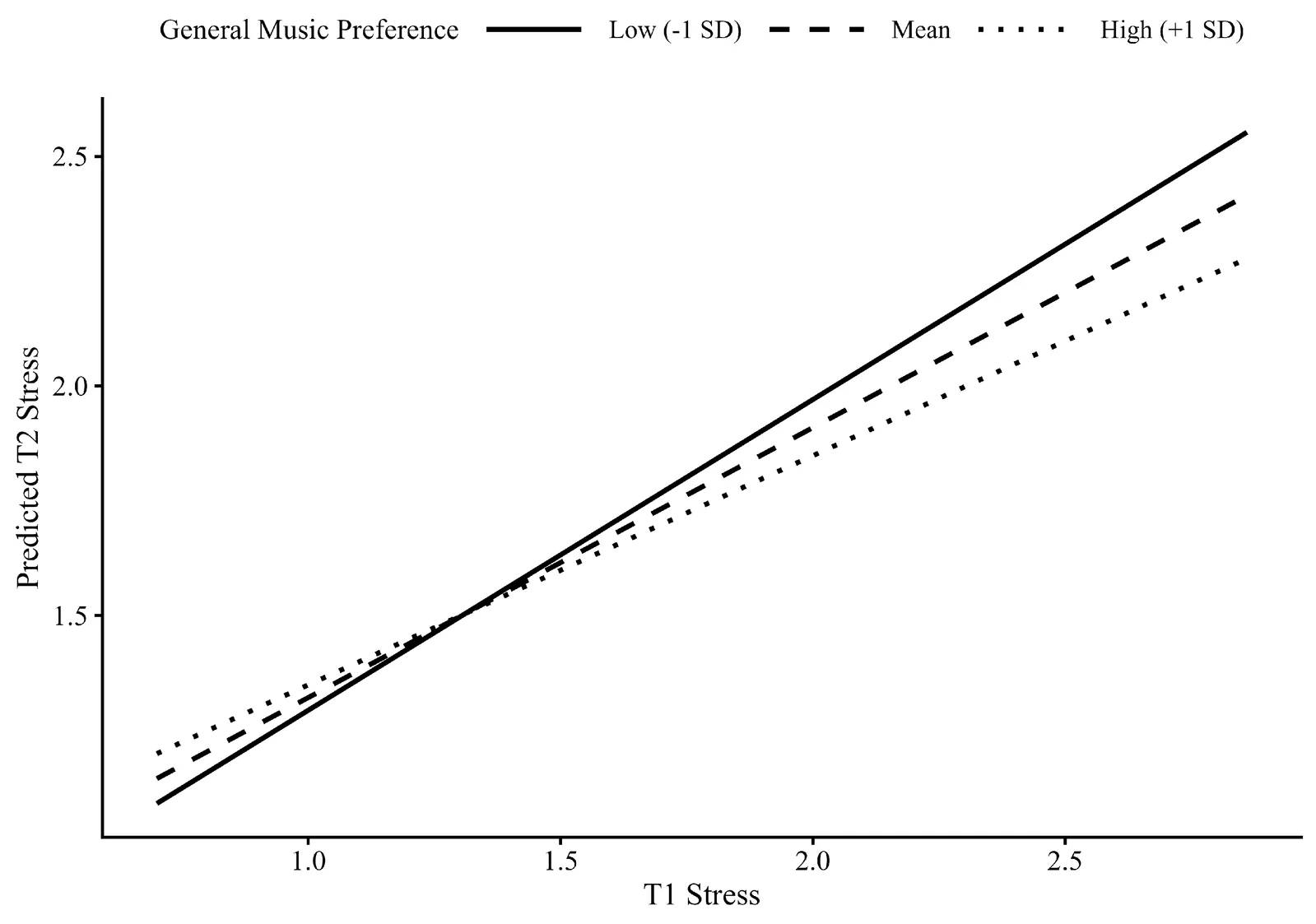
Simple slopes of the association between T1 stress and T2 stress at low, mean, and high levels of general music preference. Low and high levels represent one standard deviation below and above the mean, respectively.

However, the index of moderated mediation was not statistically significant, index = −0.0393, Boot*SE* = 0.0183, 95% BootCI [−0.0733, 0.0013], because the bootstrap confidence interval included zero. Therefore, the results did not provide statistically significant evidence that the indirect association between childhood emotional abuse and T2 stress through T1 stress varied across levels of general music preference. The significant T1 stress × general music preference interaction should be distinguished from the nonsignificant index of moderated mediation and interpreted as an exploratory full-sample finding. The results are presented in Table 2 and Table 3 and Figure 1.

### 3.3. Sensitivity Analysis Excluding Music-Major Students

To examine whether the findings were driven by music-major students, the moderated mediation model was re-estimated after excluding music-major participants. The model remained significant, *R*^2^ = 0.4398, *p* < 0.001. The direct effect of childhood emotional abuse on T2 stress was significant, β = 0.1464, *t* = 2.49, *p* = 0.0136, 95% CI [0.0305, 0.2623]. The indirect association between childhood emotional abuse and T2 stress through T1 stress remained significant at low, mean, and high levels of general music preference, with all bootstrap confidence intervals excluding zero.

However, the moderating effect of general music preference on the T1 stress–T2 stress pathway was no longer significant, β = −0.0574, *t* = −0.88, *p* = 0.3823, 95% CI [−0.1868, 0.0719]. The index of moderated mediation was also nonsignificant, Index = −0.0155, 95% BootCI [−0.0568, 0.0190]. These results indicate that, after excluding music-major students, general music preference did not significantly moderate the indirect association. Thus, the core association between childhood emotional abuse, T1 stress, and T2 stress appeared robust, whereas the music-related moderation effect was less stable and may be sensitive to sample composition.

## 4. Discussion

The present study examined whether childhood emotional abuse was associated with short-term stress continuity among university students and whether general music preference was related to variation in the T1–T2 stress pathway. Three main findings emerged. First, childhood emotional abuse was positively associated with stress at both T1 and T2. Second, T1 stress strongly predicted T2 stress, suggesting short-term stress continuity or carryover. Third, in the full sample, general music preference moderated the association between T1 stress and T2 stress, such that higher general music preference was associated with a weaker T1–T2 stress pathway. However, this moderation effect was no longer significant after excluding music-major students. Thus, the association between childhood emotional abuse and stress continuity appeared relatively robust, whereas the music-related moderation effect should be interpreted cautiously.

### 4.1. Childhood Emotional Abuse and Short-Term Stress Continuity

Consistent with previous studies, childhood emotional abuse was associated with elevated stress among university students. This finding is not presented as the primary novelty of the study, because the association between childhood emotional abuse and later psychological distress has been well documented. Recent studies among university students have shown that childhood emotional abuse or psychological maltreatment is associated with depressive symptoms, emotional distress, emotion dysregulation, and related psychological difficulties (Fan et al., 2024; Huang et al., 2025; Liu et al., 2024). The present study extends this line of work by focusing on short-term stress continuity rather than a single assessment of distress.

Students reporting higher childhood emotional abuse also reported higher T1 stress, and T1 stress was strongly associated with T2 stress. This pattern may reflect the persistence of stress among students with histories of emotional maltreatment. Emotional abuse may undermine emotional security, self-worth, and interpersonal trust, thereby increasing vulnerability to stress sensitivity, maladaptive emotion regulation, and negative self-related cognition (Alnassar et al., 2024; Huang et al., 2025; Yue et al., 2023). At a broader population level, recent evidence also indicates that childhood maltreatment contributes substantially to the burden of common mental health problems, underscoring the long-term psychological relevance of early adverse experiences (Grummitt et al., 2024).

However, the present findings should not be interpreted as evidence of a distinct causal mediation process. Because T1 and T2 stress were repeated assessments of the same construct across a three-month interval, the T1–T2 association may reflect stress persistence, rank-order stability, ongoing contextual stressors, or stable individual differences. Accordingly, the results are best understood as two-wave prospective evidence that childhood emotional abuse is associated with elevated and persistent stress among university students. This interpretation is broadly compatible with stress-sensitization perspectives, which suggest that early adversity may increase later vulnerability to stress and psychological symptoms (Bandoli et al., 2017; Starr et al., 2021). Nevertheless, the present design cannot determine whether stress continuity reflects stress sensitization, continuing environmental demands, individual differences in appraisal, or other unmeasured factors. More waves and longer follow-up intervals are needed to distinguish stable stress burden from dynamic stress change.

### 4.2. General Music Preference and the T1–T2 Stress Pathway

We also examined whether general music preference was related to variation in short-term stress continuity. In the full sample, the interaction between T1 stress and general music preference significantly predicted T2 stress. Simple slope analyses indicated that the association between T1 stress and T2 stress was weaker at higher levels of general music preference. Although the conditional indirect-effect estimates decreased descriptively from low to high levels of general music preference, the index of moderated mediation was not statistically significant because its 95% bootstrap confidence interval included zero. Therefore, the conditional indirect effects should not be interpreted as evidence of a statistically significant moderated mediation effect.

Importantly, general music preference was not significantly correlated with childhood emotional abuse, T1 stress, or T2 stress at the bivariate level. This suggests that general music preference was not simply associated with lower stress overall. Instead, its role emerged only in the conditional model, where it was related to the strength of stress continuity. In the full sample, this pattern suggested that general music preference might be related to variation in the persistence of existing stress, but it did not establish a stable buffering or protective effect.

This interpretation should be clearly distinguished from stronger claims about music listening or music-based regulation. The present study measured general liking for different music types, not actual music listening frequency, listening context, listening motives, or intentional regulation through music. Previous work indicates that music is often used for mood regulation and that music listening may be involved in emotion regulation processes (Chong et al., 2024; Saarikallio & Erkkilä, 2007). However, recent evidence also emphasizes that music-related regulation depends on listener characteristics, context, musical features, and mode of engagement (Adiasto et al., 2022; Chong et al., 2024; Morgan & Marroquín, 2024). Therefore, general music preference in the present study should be interpreted as a broad music-related individual-difference variable rather than as a direct indicator of active music-based self-regulation.

Evidence from other national contexts further suggests that the relationship between music and stress depends substantially on how, why, and under what circumstances music is used. In a German ambulatory study of university students, music listening was associated with lower perceived stress, particularly when relaxation was reported as the reason for listening (Linnemann et al., 2015). In a Swiss laboratory experiment, listening to relaxing music before an acute psychosocial stressor influenced psychobiological stress responses, although the pattern differed across physiological and subjective indicators (Thoma et al., 2013). Among Australian first-year university students, both domestic and international students rated music listening as a useful coping strategy during the COVID-19 pandemic, and the perceived effectiveness of music listening was associated with better well-being but not with lower COVID-19-related stress (Vidas et al., 2021). A large German survey similarly identified arousal and mood regulation as a major function of music listening, while distinguishing the functional use of music from stylistic preference alone (Schäfer et al., 2013). These findings from Germany, Switzerland, and Australia provide useful cross-context comparisons, but they primarily concern actual music listening, listening purposes, or regulatory functions rather than broad music preference. They therefore support the importance of examining listening motives and contexts in future research but do not directly validate the general preference effect observed in the present study.

The sensitivity analysis further supports this cautious interpretation. After excluding music-major students, the core associations among childhood emotional abuse, T1 stress, and T2 stress remained significant, but the moderating effect of general music preference was no longer significant. This suggests that the music-related moderation effect may be sensitive to sample composition. Music-major students may differ from non-music-major students in formal training, music exposure, familiarity with music categories, and the personal meaning of music preference. Although major type was controlled in the full-sample model, the sensitivity analysis indicates that statistical adjustment alone may not fully address subgroup differences.

Taken together, the significant interaction in the full sample suggests that general music preference may be related to variation in the T1–T2 stress association. However, the nonsignificant index of moderated mediation and the disappearance of the interaction after music-major students were excluded indicate that this pattern was not robust across statistical specifications. Therefore, the music-related finding should be viewed as exploratory, sample-sensitive, and hypothesis-generating rather than as evidence of a confirmed buffering effect. Cross-cultural research should directly compare listening frequency, listening motives, coping-oriented music use, and culturally specific musical engagement to determine whether and under what conditions music is associated with stress continuity.

### 4.3. Theoretical and Practical Implications

The main contribution of this study lies in its focus on short-term stress continuity following childhood emotional abuse. Rather than simply confirming that childhood emotional abuse is associated with later stress, the study suggests that students with higher childhood emotional abuse may experience more persistent stress across a short follow-up period. This distinction is important because persistent stress may represent a more enduring burden than a single assessment of distress. For university mental health services, students with histories of emotional maltreatment may require attention not only to current stress but also to whether stress remains elevated over time.

The music-related findings have more limited but potentially useful theoretical implications. The results do not support claims about specific music styles, nor do they justify treating music preference as an indicator of active self-regulation. Instead, they suggest that general music preference may be a potentially relevant individual-difference variable in stress continuity, although this effect appears sample-dependent. This interpretation is consistent with the view that music-related variables should be separated into distinct constructs, including general music liking, actual listening behavior, listening motives, contextual use, and intentional use of music for affect regulation (Chong et al., 2024; Morgan & Marroquín, 2024; Rentfrow & Gosling, 2003).

Practically, the findings do not justify recommending particular music genres as protective. They also do not show that music listening reduces stress. A more appropriate implication is that music-related variables deserve further investigation as accessible and personally meaningful aspects of students’ daily lives. Future university-based studies could examine whether students who actively use music for relaxation, mood repair, attentional redirection, or emotional processing show different stress trajectories over time.

### 4.4. Limitations and Future Directions

Several limitations should be noted. First, although we used a two-wave prospective design, the observational nature of the research precludes causal inference. Childhood emotional abuse, T1 stress, general music preference, and T2 stress were not experimentally manipulated, and unmeasured factors may have influenced the observed associations. In addition, because T1 and T2 stress were repeated assessments of the same construct over a three-month interval, the T1–T2 pathway should be interpreted as stress continuity or carryover rather than as a distinct mediating mechanism. Future research should use designs with three or more measurement waves, longer and theoretically justified follow-up intervals, and repeated assessments of intervening academic, interpersonal, and life stressors. Such designs would help determine whether stress remains stable, accumulates, fluctuates, or changes in response to new experiences. Cross-lagged, latent growth, and intensive longitudinal approaches may also help separate relatively stable individual differences from within-person changes in stress over time.

Second, the research relied on self-report measures, including retrospective reports of childhood emotional abuse, which may be affected by recall bias and common method variance. Future research should combine self-reports with behavioral indicators of emotion regulation, physiological measures of stress, ecological momentary assessment, and information from multiple sources. Daily-diary or ecological momentary designs may be particularly useful for examining whether music preference and actual music listening are associated with day-to-day changes in perceived stress. Where ethically and practically feasible, prospective records of adverse experiences may also reduce reliance on retrospective recall.

Third, the sample was drawn from a single university, and the final matched panel was reduced after attrition. Although the attrition analysis showed no significant difference between retained and lost participants in T1 stress, selective attrition on unmeasured variables cannot be ruled out. A formal a priori power analysis was not used to determine the final matched sample, which was based on the number of participants who completed both waves and could be successfully matched. However, a sensitivity power analysis indicated that, with 237 participants, the research had 80% power to detect an incremental interaction effect of approximately *f*^2^ = 0.0334 or larger. Thus, the sample was reasonably adequate for detecting interaction effects of this magnitude, although statistical power may still have been limited for smaller interaction effects and for the overall moderated mediation effect. This concern is particularly relevant to the sensitivity analysis excluding music-major students, which was based on a smaller subsample. The uneven distribution of academic years and major types also limits generalizability. Future research should conduct preregistered a priori power analyses specifically tailored to conditional process models, establish recruitment targets that account for expected attrition, and recruit larger multisite samples with more balanced representation across academic years and disciplines. Replication across universities, regions, and cultural contexts would also clarify whether the observed associations are specific to the present sample or generalize to broader student populations. Stratified recruitment or planned oversampling of music-major students may further permit adequately powered comparisons between students with different levels of musical training.

Fourth, general music preference was measured as a broad preference index adapted for Chinese university students, rather than as a fully validated measure of culturally specific music engagement or active music-based regulation. Moreover, the interaction involving general music preference was no longer significant after excluding music-major students, suggesting that this finding may be sensitive to sample composition. Future research should distinguish more clearly among general liking for music, preference for particular genres, listening frequency and duration, musical training, situational listening, and the purposeful use of music for coping or emotion regulation. Culturally grounded instruments should be developed and validated to capture both locally meaningful music categories and the functions that music serves in everyday life. Researchers could also examine whether listening motives, stress-related coping goals, perceived emotional fit, reward sensitivity, or social connectedness explain when and for whom music is associated with lower stress continuity. Comparisons between music-major and non-music-major students should be planned in advance rather than treated solely as post hoc sensitivity analyses.

Future experimental and intervention research may further test whether deliberately selected music, participant-chosen music, or structured music-based coping strategies produce changes in stress. Such research should compare these conditions with suitable nonmusical and passive-listening controls and should assess both immediate and sustained outcomes. Importantly, preference for music should not be assumed to produce regulatory benefits automatically; future work should determine whether any effects depend on actual engagement, coping intentions, listening context, and the match between the music and the listener’s emotional needs.

Because the research was not preregistered, all findings should be read as theory-informed, short-term prospective associations rather than as causal, clinical, or intervention effects. The most robust conclusion is that childhood emotional abuse was associated with elevated and persistent stress across the two-wave period, whereas the role of general music preference remains preliminary and hypothesis-generating. Accordingly, replication with larger samples, stronger temporal designs, direct measures of music use, and preregistered hypotheses is necessary before general music preference can be regarded as a reliable boundary condition of stress continuity.

## 5. Conclusions

The present study indicates that childhood emotional abuse was associated with elevated and persistent stress among university students across a three-month period. T1 stress accounted for part of the prospective association between childhood emotional abuse and T2 stress; however, because T1 and T2 represented repeated assessments of the same construct, this pathway should be interpreted as short-term stress continuity or carryover rather than as evidence of a distinct causal mediation process. In the full sample, the association between T1 stress and T2 stress varied significantly as a function of general music preference. However, the index of moderated mediation was not statistically significant, and the interaction effect was no longer significant after music-major students were excluded. Thus, the findings do not provide robust evidence that general music preference alters the indirect association between childhood emotional abuse and subsequent stress. Overall, the most consistent finding was the association of childhood emotional abuse with short-term stress persistence, whereas the role of general music preference remains preliminary, sample-sensitive, and hypothesis-generating. Future research should examine this possibility using larger and more diverse samples and more direct, culturally grounded measures of music listening behavior, motives, contexts, and regulatory use.

## Figures and Tables

**Table 1:** Spearman Correlation Matrix and Descriptive Statistics for All Variables (*N* = 237).

Variable	*M*	*SD*	1	2	3	4
1. T1 Stress	1.78	0.54	—			
2. T2 Stress	1.78	0.51	0.671 ***	—		
3. Childhood emotional abuse	6.87	2.71	0.358 ***	0.309 ***	—	
4. General music preference	4.81	0.8	−0.035	−0.039	−0.03	—

*** *p* < 0.001.

**Table 2:** Moderated Mediation Regression Analyses.

Variable	T1 Stress			T2 Stress		
	β	*SE*	*t*	β	*SE*	*t*
Constant	0.359	0.239	1.504	−0.334	0.198	−1.691
Childhood emotional abuse	0.354 ***	0.061	5.827	0.122 *	0.054	2.277
T1 stress	—	—	—	0.588 ***	0.054	10.834
General music preference	—	—	—	−0.052	0.051	−1.027
T1 stress × General music preference	—	—	—	−0.111 *	0.044	−2.54
Gender	−0.281 *	0.129	−2.176	−0.047	0.108	−0.436
Academic year	−0.122	0.103	−1.191	0.179 *	0.085	2.114
Field of study	−0.01	0.167	−0.06	−0.227	0.138	−1.64
*R* ^2^	0.144			0.427		
*F*	9.770 ***			24.410 ***		

Note. *N* = 237. Gender, academic year, and field of study were controlled. The interaction term refers to T1 stress × general music preference. * *p* < 0.05, *** *p* < 0.001.

**Table 3:** Conditional Effects and Conditional Indirect Effects.

General Music Preference	Effect of T1 Stress on T2 Stress	*SE*	*t*	95% CI	Conditional Indirect Effect	Boot*SE*	95% Boot CI
Low (−1 *SD*)	0.678 ***	0.073	9.61	[0.556, 0.843]	0.248	0.055	[0.154, 0.370]
Mean	0.588 ***	0.054	10.834	[0.481, 0.695]	0.208	0.045	[0.132, 0.308]
High (+1 *SD*)	0.499 ***	0.067	7.174	[0.346, 0.608]	0.169	0.042	[0.100, 0.265]
Index of moderated mediation					−0.039	0.018	[−0.073, 0.001]

Note. Conditional indirect effect refers to childhood emotional abuse → T1 stress → T2 stress at different levels of general music preference. Bootstrap confidence intervals were based on 5000 resamples. Because the confidence interval for the index of moderated mediation included zero, the moderated mediation effect should be interpreted cautiously. *** *p* < 0.001.

## Data Availability

The data that support the findings of this study are available from the corresponding author upon reasonable request due to privacy.

## Supplementary Materials

The following supporting information can be downloaded at https://www.mdpi.com/article/10.3390/bs16091499/s1: File S1: General Music Preference Measure adapted for Chinese university students.
