# Supplementary material for: Childhood Emotional Abuse and Short-Term Stress Continuity Among University Students: The Role of General Music Preference
Source: Behav Sci (Basel). 2026 Aug 26;16(9):1499. doi: 10.3390/bs16091499 (PMC13603463; doi:10.3390/bs16091499)
Supplement: Supplementary file 1 [file behavsci-16-01499-s001.zip › File S1. General Music Preference Measure.pdf]

Instructions

Dear students,

This questionnaire aims to understand university students' preferences for different types of music. Several common music genres are listed below, together with their core features and typical examples to help you better understand each category. Please indicate how much you personally like each type of music based on your genuine feelings.

Please rate each item on a 1–7 scale:

1 = Strongly dislike
2 = Moderately dislike
3 = Slightly dislike
4 = Neutral
5 = Slightly like
6 = Moderately like
7 = Strongly like

There are no right or wrong answers. You do not need to be familiar with all the example songs or artists. Please respond according to your overall impression of each music genre and your personal preference. If you are not very familiar with a particular genre, you may refer to its core features and typical examples and choose the option that best reflects your feeling.

Thank you for your participation.

No.	Music Genre	Core Features	Typical Examples Familiar to Post-2000s University Students	Preference Rating (1–7)
1	Alternative/Indie	Distinctive, niche, creative, introspective, unconventional	Accusefive, No Party for Cao Dong, Summer Invasion Project, deca joins	1 2 3 4 5 6 7
2	Rhythm and Blues (R&B)	Smooth, rhythmic, sensual, urban, romantic	Tia Ray, Khalil Fong, ØZI, MaSiWei melodic rap	1 2 3 4 5 6 7
3	Classical/Light Music	Elegant, profound, grand, peaceful, narrative	Game/anime soundtracks such as Yu-Peng Chen's music for Genshin Impact, Lang Lang, healing instrumental music	1 2 3 4 5 6 7
4	Country/Folk	Simple, narrative, heartfelt, pastoral, sincere	Mao Buyi narrative songs, Chen Hongyu, Good Meimei Band,	1 2 3 4 5 6 7

			early Taylor Swift	
5	Electronic/Dance	Dynamic, trendy, psychedelic, energetic, technological	CORSAK Hu Mengzhou, KSHMR, Jolin Tsai's "Play," virtual idol songs such as "Ievan Polkka"	1 2 3 4 5 6 7
6	Urban Folk/Pop Folk	Fresh, warm, relatable, melodic, youthful	Yan Renzhong, Shen Yicheng, Xieyou Shaoshuai, Zhao Lei's "Chengdu"	1 2 3 4 5 6 7
7	Heavy/Core Music	Explosive, cathartic, powerful, rebellious, impactful	Nine Treasures folk metal, Arch Enemy melodic death metal	1 2 3 4 5 6 7
8	Rap/Hip-Hop	Attitudinal, rhythmic, cool, authentic, street-oriented	Wang Yitai, Key.L Liu Cong, Jiang Yunsheng, Pharaoh, Higher Brothers	1 2 3 4 5 6 7
9	Jazz/Urban Pop	Laid-back, swinging, refined, ambiguous, sophisticated	Lü Yanliang, Laufey, Orange Ocean selected works, J.Sheon	1 2 3 4 5 6 7
10	Chinese/Western Pop	Catchy, mainstream, diverse, memorable, star-oriented	Jay Chou, JJ Lin, G.E.M., Billie Eilish, Justin Bieber	1 2 3 4 5 6 7
11	Chinese Style/Ancient-Style Music	Poetic, melodious, atmospheric, Eastern, story-based	Interestingcn Music, Hetu, Yinlin, selected works by Zhou Shen, "Chi Ling"	1 2 3 4 5 6 7
12	Pop Rock/Band Music	Passionate, energetic, band-based, live-performance-oriented, youthful	Mayday, GALA, New Pants, Imagine Dragons	1 2 3 4 5 6 7
13	Soul/Funk	Soulful, free, retro, joyful, groovy	Tia Ray, Khalil Fong, Bruno Mars' "Uptown Funk"	1 2 3 4 5 6 7
14	Film/Game/Anime Soundtracks	Visual, atmospheric, narrative, immersive, memory-evoking	"Lonely Warrior" from League of Legends, "Su," theme song from Someday or One Day, RADWIMPS' music for Your Name	1 2 3 4 5 6 7
